# Solid Thin-Film Battery Using a Densely Packed LiNi_0.5_Mn_1.5_O_4_ Crystal Layer

**DOI:** 10.1021/acsomega.4c09393

**Published:** 2025-04-18

**Authors:** Shigeru Kobayashi, Nobuyuki Zettsu, Kazunori Nishio, Ryota Shimizu, Toshiki Imabori, Yoshiki Saito, Katsuya Teshima, Taro Hitosugi

**Affiliations:** † Department of Chemistry, The University of Tokyo, Tokyo 113-0033, Japan; ‡ Department of Materials Chemistry, Shinshu University, Nagano 380-8553, Japan; § Research Institute for Supra-Materials, Shinshu University, Nagano 380-8553, Japan; ∥ Energy Landscape Architectonics Brain Bank, Shinshu University, Nagano 380-8553, Japan; ⊥ School of Materials and Chemical Technology, Institute of Science Tokyo, Meguro, Tokyo 152-8552, Japan

## Abstract

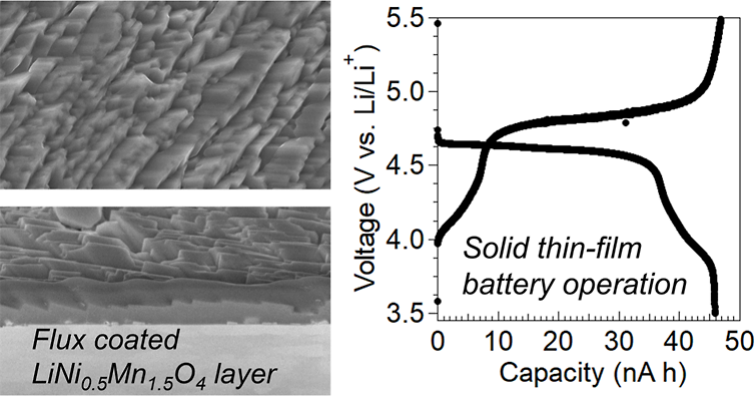

A 5 V-class LiNi_0.5_Mn_1.5_O_4_ (LNMO) positive electrode has excellent potential for increasing the energy density of solid-state Li batteries. In this study, we report the stable cycling of a solid thin-film Li battery using a densely packed LNMO crystal layer, which has a high packing density and a well-developed crystal plane. Annealing a battery reduces the resistance at the interface of a Li_3_PO_4_ solid electrolyte and LNMO for an order of magnitude, from 5.3 × 10^2^ to 5.7 × 10^1^ Ωcm^2^, enhancing the battery performance. This paper lays the groundwork for developing all-solid-state Li batteries using densely packed LNMO layers.

## Introduction

All-solid-state Li batteries attract substantial interest because of the increasing demand for rechargeable batteries with higher energy and power density.^1^ Spinel LiNi_0.5_Mn_1.5_O_4_ (LNMO) positive electrode has garnered considerable attention for all-solid-state Li batteries owing to its high redox voltage of ∼4.7 V vs Li/Li+.^2,3^ A densely packed layer synthesized using the flux coating method^4,5^ is one of the promising positive electrode layers because of the high electrode density. The densely packed layer is a polycrystalline film composed of idiomorphic single-crystal particles; the surface structures and crystal orientations can be controlled by tuning the fabrication conditions (Figure S1a).^6^ Furthermore, due to its capability to achieve a thickness at the micrometer scale, the densely packed layer holds promise for potential applications in bulk all-solid-state batteries. Those densely packed layers have already shown their great potential as positive electrodes for batteries using liquid electrolytes.^7,8^ However, there has been no report on the all-solid-state Li battery or even solid thin-film batteries using the densely packed layers. Therefore, the solid thin-film battery operation with a densely packed LNMO layer (hereafter called a dense-LNMO layer) is highly desired.

One of the reasons hindering the performance is the high resistance at the interface of the solid electrolyte and the dense-LNMO layer (interface resistance).^9^ Therefore, it is critical to reduce the interface resistance. We have recently demonstrated the drastic reduction of the Li_3_PO_4_ (solid electrolyte)–LiCoO_2_ interface resistance values by annealing an entire thin-film battery (hereafter called a recovery annealing).^10^ The recovery annealing of a thin-film battery at 150 °C reduced interface resistance from ∼140 to ∼10 Ωcm^2^. This low interface resistance is approximately half that of the typical value of a Li-ion battery using a liquid electrolyte.^11^ The key factor in reducing the interface resistance is to suppress the proton density near the surface of LiCoO_2_; the protons are supplied from H_2_O in the ambient. This recovery annealing may be effective for improving the performance of batteries using other electrode materials. Although the impact of the recovery annealing for LiCoO_2_ thin films has been reported, there is no report for 5 V-class electrode material LNMO. Furthermore, it remains unclear whether the recovery annealing can be effective not only in flat thin films with a single orientation but also in dense-LNMO layers with multiple orientations and a rough surface.

In this paper, we report the solid thin-film Li battery operation using dense-LNMO layers. First, we show that the recovery annealing also applies to batteries consisting of LNMO (001) epitaxial thin films,^12−14^ suggesting that the proposed mechanism is universal for other oxide electrode materials. The recovery annealing reduces the Li_3_PO_4_–LNMO (001) interface resistance by an order of magnitude, leading to an improvement in the capacity, charge–discharge characteristics, and cyclic performance. Next, we investigate the performance of batteries using a dense-LNMO layer. The recovery annealing also reduces the resistance at the interface of Li_3_PO_4_ and idiomorphic dense-LNMO layer by almost an order of magnitude, leading to stable battery cycling performance for at least 30 cycles. This research paves the way for applying a dense-LNMO layer to bulk-type all-solid-state Li batteries.

## Experimental Section

### Fabrication of a Dense-LiNi_0.5_Mn_1.5_O_4_ Layer

Dense-LNMO layers (thickness of ∼2 μm) were directly prepared on a Pt substrate by mediated chemical vapor deposition using LiCl–KCl mixed molten salt, promoting facet-oriented crystal growth (flux coating). The crystallization processes were controlled via changes in the level of supersaturation. When a saturated solution volatilizes, it becomes supersaturated. Crystals nucleated while the vaporization continued at a designated temperature. Subsequently, the growth of LNMO crystals with well-defined shapes was promoted on the Pt substrate at the same temperature because of the continuous volatilization of molten salts.

Reagent-grade LiCl (8.908 g), NiNO_3_·6H_2_O (1.137 g), MnNO_3_·6H_2_O (2.721 g), and KCl (10.663 g) powders (Wako Pure Chemical Industries, Ltd.) were used as the starting materials for the dense-LNMO layers. The mixture ratio of NiNO_3_·6H_2_O and MnNO_3_·6H_2_O was 1:3 at mol % and LiCl/(NiNO_3_·6H_2_O)+(MnNO_3_·6H_2_O) = 15.7. LiCl was used as both the Li source and the flux. Pt substrates with a diameter of 14 mm were used for crystal growth and as current collectors for battery assembly. The Pt substrate surfaces were washed with water and subsequently dried at 600 °C prior to use. A schematic of the flux coating is presented in Figure S1. A mixture of solute and flux was placed in a 30 cm^3^-capacity alumina crucible. A Pt substrate was placed flat over the crucible using an alumina support. The size and shape of the support were designed considering the airflow because all raw materials were vaporized. The distance between the surface of the mixed raw materials and the Pt substrate was approximately 30 mm. The crucible was placed in an electric furnace heated to 700 °C at a rate of 50 °C·min^–1^ in ambient air. As soon as the crucible reached 700 °C, it was cooled to 350 °C at a rate of 200 °C·h^–1^. The dense LNMO films were cleaned with distilled water with a homogenizer to remove the remaining flux. The LNMO electrodes were dried at 100 °C under a dry atmosphere. Finally, the LNMO electrodes were annealed at 700 °C for 24 h in the air to enhance crystallinity and Ni/Mn ordering.

### Battery Fabrication

Two types of solid thin-film batteries are fabricated in this study: (1) thin-film battery using epitaxial LNMO film and (2) battery using a dense-LNMO layer. The schematics of each battery fabrication process are described in Figure S1b.

1 Thin-film battery using an epitaxial LNMO film: The thin-film batteries consisted of a LaNiO_3_ current collector (thickness of 20 nm), LNMO positive electrode (thickness of 60 nm), Li_3_PO_4_ (LPO) solid electrolyte (thickness of 1 μm), and Li metal negative electrode (thickness of 1 μm, 0.5 mm diameter). Apart from the exposure of the LNMO surface to the air ambient, the steps in the fabrication of films were a all-in-vacuum (in vacuo) process.^15^ A stack of thin films was deposited on a 0.5 wt % Nb-doped SrTiO_3_(100) substrate. LaNiO_3_ and LNMO thin films were deposited by pulsed laser deposition (PLD) using a KrF excimer laser, and the LPO film was deposited by PLD using an ArF excimer laser. Then, a Li metal film was thermally evaporated. The detailed fabrication process is described elsewhere.^2^ The surface of the LNMO positive electrode was exposed to ambient air for 60 min at room temperature of approximately 25 °C. Subsequently, the films were placed into a vacuum chamber again, and LPO and Li were deposited. The active region of the thin-film batteries is defined by the area of the Li negative electrode (diameter of 0.5 mm). The recovery annealing conditions were 150 °C and 60 min in a vacuum (<1 × 10^–4^ Pa). The recovery-annealed battery was annealed before the first cycle.
2 Battery using a dense-LNMO layer: A Li_3_PO_4_ solid electrolyte (thickness of 4 μm) was deposited on a dense-LNMO layer using radio frequency (RF) magnetron sputtering (RF output power of 100 W, Ar gas partial pressure of 0.15 Pa). A 2-in. Li_3_PO_4_ target (Toshima Manufacturing Co., Ltd., Japan) was used for the deposition. Subsequently, a Li negative electrode (thickness of 2 μm, diameter of 0.5 mm) was deposited using the thermal evaporation method. Both Li_3_PO_4_ and Li were deposited on unheated samples.

### Characterization of Thin Films and Battery Performances

The thicknesses and surface roughness of the thin films were determined using a stylus profiler (Veeco, Dek-Tak 150). The cross-section image of a thin LNMO film was characterized on the Au layer using field-emission scanning electron microscopy (FE-SEM, Hitachi, S-5500) with an acceleration voltage of 10 kV. The surface and cross-sectional morphologies of the as-grown flux-synthesized dense-LNMO layers were characterized using FE-SEM (JEOL, JSM-7600F) with an acceleration voltage of 15 kV. The crystal structures were determined using X-ray diffraction with a Cu–Kα radiation source (RIGAKU, MiniflexII).

The battery performances of the solid thin-film Li batteries were evaluated using cyclic voltammetry (CV), constant current (CC) charge–discharge, and AC impedance spectroscopy (frequency ranged from 10^–1^ to 10^6^ Hz, 30 mV amplitude) inside an Ar-filled glovebox (H_2_O < 0.25 ppm) using a potentiostat/galvanostat with a frequency response analyzer (Biologic SAS, SP-150 and VMP3). The interface area for calculating the interface resistance value is defined with the battery cross-sectional area (diameter of 0.5 mm).

To evaluate the fundamental electrochemical characteristics of the dense-LNMO layers, we performed galvanostatic charge–discharge tests using an R2032-type coin cell. All dense-LNMO layers were dried at 120 °C in a vacuum oven prior to cell assembly. Li metal foil and a porous polypropylene film (#2500; Celgard, Charlotte) were used as the counter electrode and separator, respectively. A solution of 1 M LiPF_6_ in a mixture of ethylene carbonate and dimethyl carbonate (1:1 vol %) was used as the electrolyte. The coin-type cells were assembled in an Ar-filled glovebox (MDB-2BL; Miwa Mfg) under a controlled atmosphere containing 0.1 ppm of H_2_O and O_2_. Galvanostatic charge–discharge tests were performed using a potentiostat/galvanostat (HJ1020Msd8; Hokuto Denko). The cutoff voltage range for the battery tests was controlled between 3.5 and 4.8 V vs Li/Li^+^. All electrochemical measurements were performed at a room temperature of approximately 23 °C.

## Results and Discussion

**Figure 1:**
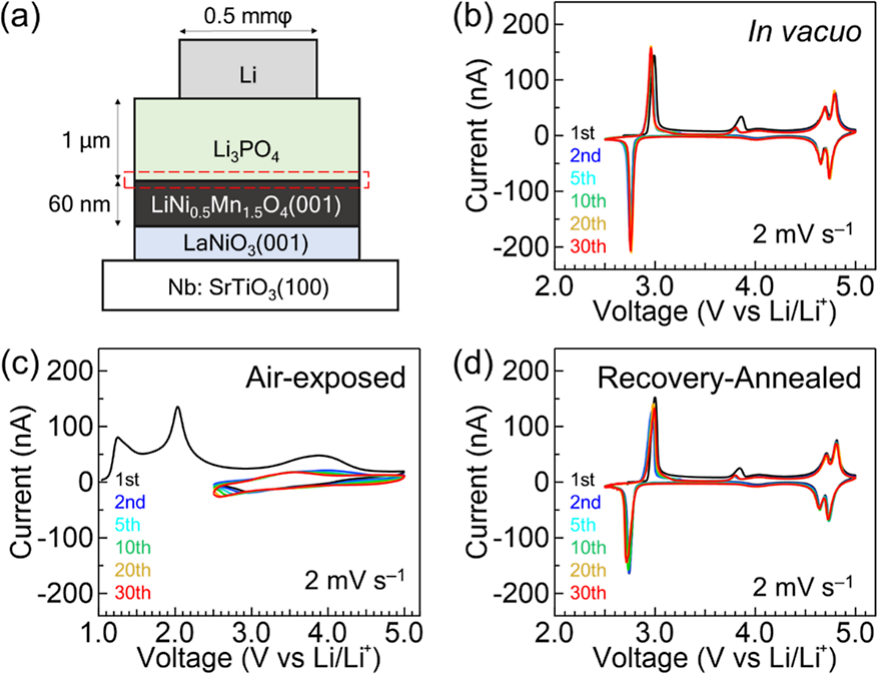
(a) Schematic of a fabricated thin-film battery using a LNMO epitaxial thin film. The thickness of the LNMO epitaxial thin film is approximately 60 nm. The interface of Li_3_PO_4_ and LNMO is the focus of this study (red dotted line). (b–d) Cyclic voltammetry curves of (b) in vacuo, (c) air-exposed, and (d) recovery-annealed batteries. The sweep rate was 2 mV s^–1^.

First, we confirm the effect of a recovery annealing to the interface of Li_3_PO_4_ and LNMO (001) epitaxial film. LNMO (001) is suitable for investigating the impact of recovery annealing because it has been reported that the surface degradation due to atmospheric gases is most significant in the (001)-exposed crystal facet.^15^ The methods for the fabrication of LNMO (001) epitaxial films are shown in the Supporting Information and elsewhere.^12^ We compared three types of thin-film batteries (Figure 1a) with different fabrication processes; (1) the LNMO surface and other interfaces were never exposed to air (in vacuo battery),^16^ (2) the LNMO surface was exposed to air (air-exposed battery); then, Li_3_PO_4_ and Li were deposited at room temperature to form a battery, and (3) the air-exposed battery was annealed at 150 °C in a vacuum of ∼1 × 10^–5^ Pa for 60 min (recovery-annealed battery).

Figure 1b–d shows the cyclic voltammetry (CV) voltammograms of the three types of thin-film batteries. The in vacuo battery shows clear current peaks originating from the redox reactions between LiNi_0.5_Mn_1.5_O_4_ and Li_0_Ni_0.5_Mn_1.5_O_4_ at around 4.7 V vs Li/Li^+^ (Figure 1b).^17,18^ In addition, redox peaks indicating the transition between Li_2_Ni_0.5_Mn_1.5_O_4_ and Li_1_Ni_0.5_Mn_1.5_O_4_ are observed at lower voltages (2.7–3.0 V vs Li/Li^+^).^12^ In contrast, the air-exposed battery exhibits no current peak in the CV curves (Figure 1c). This result indicates that exposing the LNMO surface to air significantly degrades the surface of LNMO and leads to poor battery performance.

We confirmed that the recovery annealing improves the battery performance, as observed for thin-film batteries using LiCoO_2_. Sharp current peaks appear in the CV curves of the recovery-annealed battery (Figure 1d). Surprisingly, the peak current values are comparable to those of the in vacuo battery. In addition, the peak separation between oxidation and reduction reactions is ∼0.07 V, which is identical to that of in vacuo batteries. This small peak separation indicates that annealing significantly reduces the internal resistance of the battery. The discharge capacities, measured in the voltage range of 4.5–5.0 V vs Li/Li^+^ for low (2 μA cm^–2^) and high (1 mA cm^–2^) current densities, were 0.87 and 0.37 μA h cm^–2^, respectively. These values are comparable to those observed in the in vacuo battery (0.87 and 0.46 μA h cm^–2^, respectively). Therefore, charge–discharge characteristics also improved (Figure S2).

**Figure 2:**
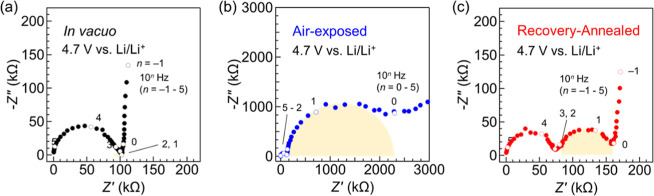
(a–c) Nyquist plots obtained at 4.7 V vs Li/Li^+^ for (a) in vacuo, (b) air-exposed, and (c) annealed batteries using a LNMO epitaxial thin film. Each open circle shows the point at each frequency (10^0^–10^5^ Hz). Yellow semicircles depict the impedance component originating from the Li_3_PO_4_–LNMO interface.

We quantitatively evaluated the impact of annealing on the interface resistances. The detailed analysis procedures, including an equivalent circuit model, are presented in Figure S3. The in vacuo battery exhibits a minimum interface resistance of 11.2 Ωcm^2^ at 4.7 V vs Li/Li^+^ (Figure 2a), consistent with the earlier report.^12^ The corresponding interface resistance of the air-exposed battery increases significantly to 4.7 × 10^3^ Ωcm^2^ at 4.7 V vs Li/Li^+^ (Figure 2b).

The recovery annealing of the air-exposed battery reduces the interface resistance from 4.7 × 10^3^ Ωcm^2^ to 1.8 × 10^2^ Ωcm^2^ at 4.7 V vs Li/Li^+^ (Figure 2c). Notably, the interface resistance decreases by an order of magnitude. Taken together, the recovery annealing is effective in reducing the Li_3_PO_4_–LNMO (001) interface resistance. We speculate that a mechanism similar to Li_3_PO_4_–LiCoO_2_ interfaces is responsible for the reduction; protons originating from adsorbed H_2_O molecules at the interface induce structural transformation, leading to a high interface resistance.^10,19^ A higher annealing temperature would be needed to further reduce the interface resistance in the Li_3_PO_4_–LNMO case.

Next, we discuss the performance of the battery using a dense-LNMO layer. Figure S4a shows the X-ray diffraction (XRD) pattern of a dense-LNMO layer on a Pt substrate. All significant diffraction peaks are assigned to Pt, LNMO, and Li_0.64_Pt_3_O_4_. The origin of the formation of Li_0.64_Pt_3_O_4_ is discussed in the Supporting Information (Figure S4). The out-of-plane XRD patterns strongly indicate that the dense-LNMO layer is (311) and (110) preferentially oriented polycrystalline film (Figure S4a). The SEM image shows the tilted crystal surfaces from the (111) plane (Figure S4b,c). The thickness of the layer is approximately 2 μm, confirmed by the cross-sectional scanning electron microscopy image (Figure S4d).

**Figure 3:**
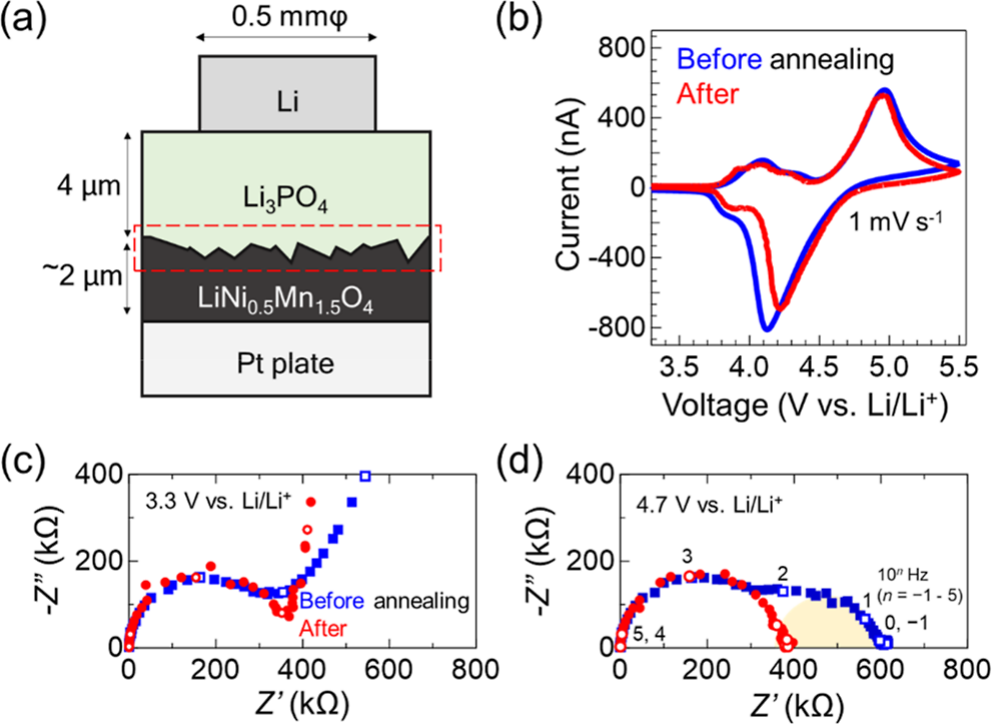
(a) Schematic of the fabricated solid thin-film battery using a flux-coated dense LNMO crystal layer. Active region of the battery is defined by a Li negative electrode area (0.5 mmφ). (b) Cyclic voltammogram of batteries using the dense-LMNO layer with (blue) and without recovery annealing (red). Recovery annealing was performed at 150 °C for 4 h in an Ar-filled glovebox. (c) and (d) show Nyquist plots obtained from the batteries at 3.3 and 4.7 V vs Li/Li^+^, respectively. Each open circle corresponds to the point at each frequency (10^0^–10^5^ Hz). Yellow semicircle in the low-frequency region (10^0^–10^2^ Hz) depicts the impedance component originating from the Li_3_PO_4_ and LNMO interface.

The dense-LNMO layers were transferred through the air and introduced into a vacuum chamber (<1 × 10^–5^ Pa). Then, without cleaning the sample surface in the vacuum chamber, Li_3_PO_4_ and Li were deposited at room temperature to form a battery (hereafter called an as-prepared battery, Figure 3a). Because the samples were transferred through ambient air, the surface of the dense-LNMO layer was contaminated with H_2_O, organic carbon species, and carbon dioxide. The thickness of Li_3_PO_4_ was increased to 4 μm to fully cover the surface of the rough dense-LNMO layer (root-mean-square surface roughness is 216 nm over 10 μm square area). The typical open-circuit voltage of the fabricated battery was 2.9 V vs Li/Li^+^.

The as-prepared battery operates without the recovery annealing (Figure 3b). This result, at sight, contrasts with the degraded performance observed for the air-exposed thin-film battery using an LNMO (001) epitaxial film (Figure 1c). However, the two results are consistent because degradation by air exposure is only limited to the surface of the dense-LNMO layer, causing the increase in the interface resistance. In the case of LiCoO_2_, only 20 to 30 nm from the surface is degraded.^18^ The thin-film battery with a LiCoO_2_ thickness over 40 nm shows redox reactions;^18^ however, the performance is limited because of the high interface resistance.

Indeed, a high interface resistance is observed in the as-prepared battery. We evaluated the Nyquist plots of discharged (3.3 vs Li/Li^+^, Figure 3c) and charged (4.7 V vs Li/Li^+^, Figure 3d) states. The semicircles (10^2^–10^5^ Hz) observed at 3.3 V vs Li/Li^+^ (discharged state) originate from the bulk ionic conduction resistance of the Li_3_PO_4_ solid electrolyte.^20^ The evaluated ionic conductivity of Li_3_PO_4_ is 6.0 × 10^–7^ S cm^–1^, comparable to that observed in previous studies.^21,22^ At 4.7 V vs Li/Li^+^ (charged state), two semicircles are observed. The frequency range of the semicircles (10^2^–10^5^ Hz) is the same as the one observed at the discharged state. The other semicircle observed at 10^0^–10^2^ Hz originates from the interface resistance. The interface resistance at 4.7 V vs Li/Li^+^, evaluated from the semicircle in the lower frequency region, is 5.3 × 10^2^ Ωcm^2^.

We next fabricated a recovery-annealed battery. The dense-LNMO layer was introduced into the vacuum chamber, followed by the deposition of Li_3_PO_4_ and Li. The battery was annealed at 150 °C in an Ar-filled glovebox before the first charging. The CV curves of the recovery-annealed battery are shown in Figure 3b. The separation of oxidation and reduction current peaks is reduced from 0.84 to 0.74 V. The result suggests a decrease in the internal resistance and a decreased battery capacity. As expected, recovery annealing reduces the interface resistance by almost 1 order of magnitude, from 5.3 × 10^2^ Ωcm^2^ to 5.7 × 10^1^ Ωcm^2^ (Figure 3d). We note that the battery’s capacity before annealing is similar to or higher than after recovery annealing. The Nyquist plot shows that the major component of the internal resistance is the Li_3_PO_4_ solid electrolyte for both before and after annealing. Because of the high resistance of the Li_3_PO_4_ solid electrolyte, no apparent difference in battery performance is observed. When reducing the thickness of the solid electrolyte layer or utilizing a solid electrolyte with high ionic conductivity, the impact of the reduction in interfacial resistance will become more pronounced.

We compare the battery characteristics of thin-film LNMO and dense-LNMO batteries. The dense structure of both thin-film LNMO and dense-LNMO layers is confirmed by cross-sectional SEM images (Figures S4d and S5). No significant difference in the bulk density is expected. Interface resistance between Li_3_PO_4_ and thin-film LNMO is observed to be 1 order of magnitude larger than that of the dense-LNMO layer following exposure to air (Figures 2a,b,d). The difference is primarily attributed to the influence of the surface orientations. LNMO thin films in this study are (001) oriented, while the dense-LNMO layer has mainly (111) crystal facets. A previous study indicated that the (100)-exposed crystal facets of LNMO degrade more easily by atmospheric gases than (111)-exposed ones.^15^ The reduction rate of interfacial resistance by annealing was similar for thin-film LNMO and dense-LNMO batteries.

**Figure 4:**
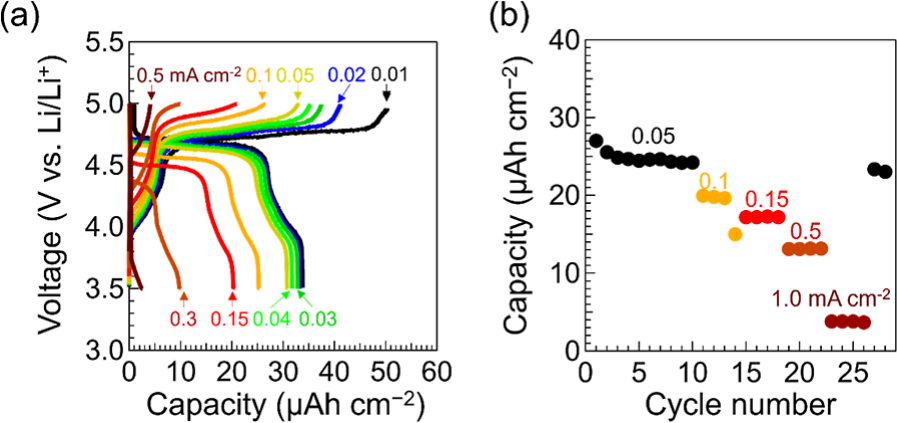
(a) Charge–discharge curves at a variety of current densities for the solid thin-film battery using a dense-LMNO layer. (b) Cyclic performance of discharge capacity at a variety of current densities.

The annealed battery shows stable charge–discharge performance for at least 30 cycles (Figure 4a). A voltage plateau, originating from the redox responses of Ni^2+/4+^ (∼4.7 V vs Li/Li^+^) is observed in the galvanostatic charge–discharge curves at a current density of 0.01 mA cm^–2^. The discharge capacity is ∼35 μA h cm^–2^ (66 nA h) for a current density of 0.01 mA cm^–2^. The corresponding utilization ratio of the dense LNMO is ∼20%. The value was calculated with the approximate thickness of 2 μm, theoretical mass density of 5.86 g cm^–3^, and capacity of 147 mA h g^–1^. The sharp grain edge geometry may cause some negative effects, such as electric field concentration leading to the inhomogeneous Li diffusion. Capacity retentions are 75% (∼26 μA h cm^–2^) for 0.1 mA cm^–2^ and 30% (∼10 μA h cm^–2^) for 0.5 mA cm^–2^. The original discharge capacity maintains 86% after 30 cycles (Figure 4b). The charge–discharge curves are in good agreement with the battery performance of the Li-ion battery consisting of a dense-LNMO layer and an organic liquid electrolyte (Figure S6). Therefore, a solid thin-film Li battery using a dense-LNMO layer is demonstrated.

## Conclusions

In summary, we reported the stable operation of a solid thin-film Li battery based on a densely packed LNMO layer. The recovery annealing reduces the interface resistance by an order of magnitude. For thin-film batteries, which are strongly influenced by interface conditions, battery properties are greatly improved by annealing. We demonstrated that the recovery is not unique to LiCoO_2_ by showing the reduction of interfacial resistance even for LNMO. These findings indicate that recovery annealing is effective at interfaces formed by various oxide electrodes and solid electrolytes. The study paves the way for all-solid-state batteries based on densely packed LNMO layers and for the understanding of the phenomena at the solid–solid interfaces.

## Supporting Information Available

Fabrication procedure of materials and solid thin-film batteries, schematics of the flux-coating process, characterization of fabricated layers, battery performance of thin-film batteries and liquid battery cell, and detailed analysis model (PDF)
