# Supplementary material for: Solid Thin-Film Battery Using a Densely Packed LiNi0.5Mn1.5O4 Crystal Layer
Source: ACS Omega. 2025 Apr 18;10(16):16073–8. doi: 10.1021/acsomega.4c09393 (PMC12044454; doi:10.1021/acsomega.4c09393)
Supplement: Supplementary file 1 — ao4c09393_si_001.pdf [file ao4c09393_si_001.pdf]

SUPPORTING INFORMATION

Solid thin-film battery using a densely-packed $LiNi_{0.5}Mn_{1.5}O_4$ crystal layer

Shigeru Kobayashi[1*], Nobuyuki Zettsu[2, 3, 4**], Kazunori Nishio[5], Ryota Shimizu[1], Toshiki Imabori[2], Yoshiki Saito[2], Katsuya Teshima[2, 3], Taro Hitosugi[1***]

[1] Department of Chemistry, The University of Tokyo, 113-0033 Tokyo, Japan
[2] Department of Materials Chemistry, Shinshu University, Nagano, 380-8553 Japan.
[3] Research Institute for Supra-Materials, Shinshu University, Nagano, 380-8553 Japan
[4] Energy Landscape Architectonics Brain Bank, Shinshu University, Nagano, 380-8553 Japan
[5] School of Materials and Chemical Technology, Institute of Science Tokyo, Meguro, Tokyo 152-8552, Japan

*kobayashi-shigeru@g.ecc.u-tokyo.ac.jp
**zettsu@shinshu-u.ac.jp
***hitosugi@g.ecc.u-tokyo.ac.jp

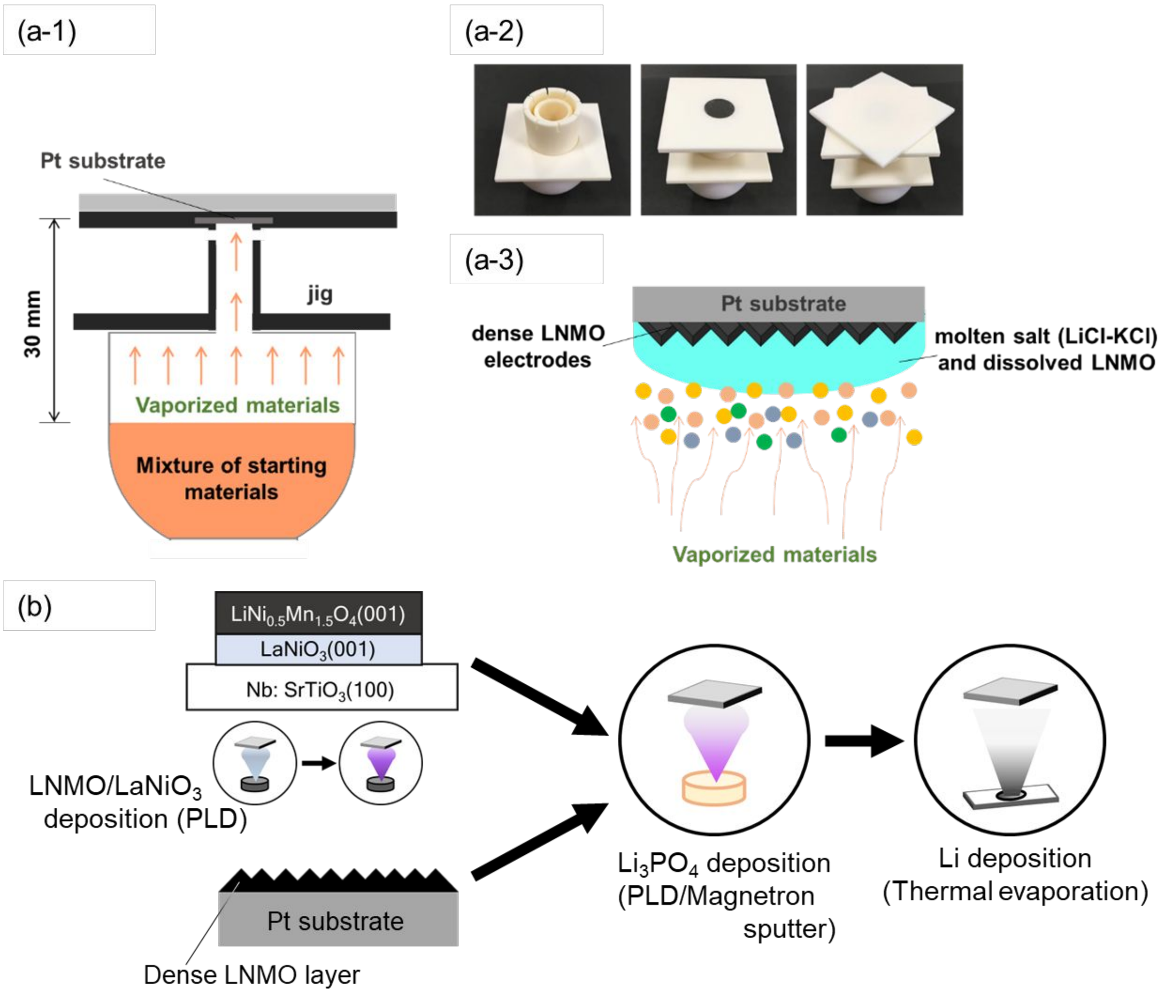

Figure S1. Schematic illustration of the $LiNi_{0.5}Mn_{1.5}O_4$(LNMO) battery fabrication process. (a-1) Schematic of the experimental setup for the dense-LNMO crystal layer preparation, (a-2) corresponding photo images, and (a-3) plausible illustrated scheme of LNMO crystal layer formation. (b) Schematic of the battery fabrication processes using epitaxial LNMO thin film and dense LNMO crystal layer.

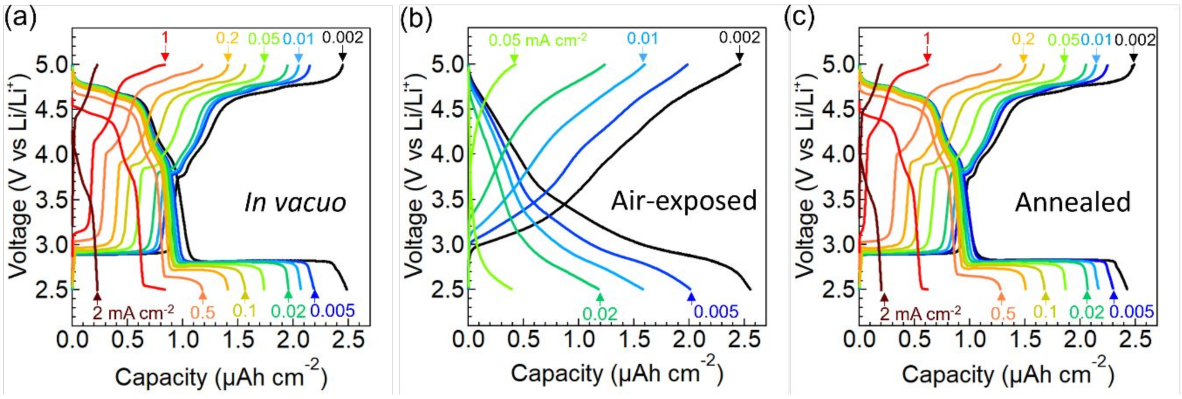

Figure S2. Charge–discharge curves of thin-film batteries using $LiNi_{0.5}Mn_{1.5}O_4$ (LNMO) thin films. This figure shows charge-discharge curves obtained from thin-film batteries using 60-nm-thick LNMO positive electrodes: (a) *in vacuo*, (b) air-exposed, and (c) recovery-annealed.

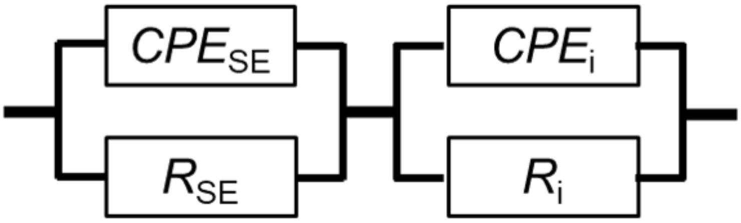

Figure S3. Estimation of the interface resistance using an equivalent circuit. The ionic conductivity of the Li_3PO_4 (LPO) solid electrolyte and interfacial resistances at 4.7 V vs. Li^+/Li were estimated by fitting the obtained impedance spectra with the equivalent circuit model depicted in Fig. S4. R_{SE} and CPE_{SE} represent the resistance and capacitance of the LPO solid electrolyte, respectively. R_i and CPE_i indicate the resistance and constant phase element (CPE) of the interface between the LPO solid electrolyte and $LiNi_{0.5}Mn_{1.5}O_4$ positive electrode, respectively. Q_i and a_i are the CPE interfacial parameters. Ideally, the ionic conduction resistance would be represented by a parallel circuit including capacitance and resistance, but we used the CPE because the circuit did not fit the interfacial element. The interfacial resistance was calculated by normalizing R_i for the active area of 0.196 mm^2.

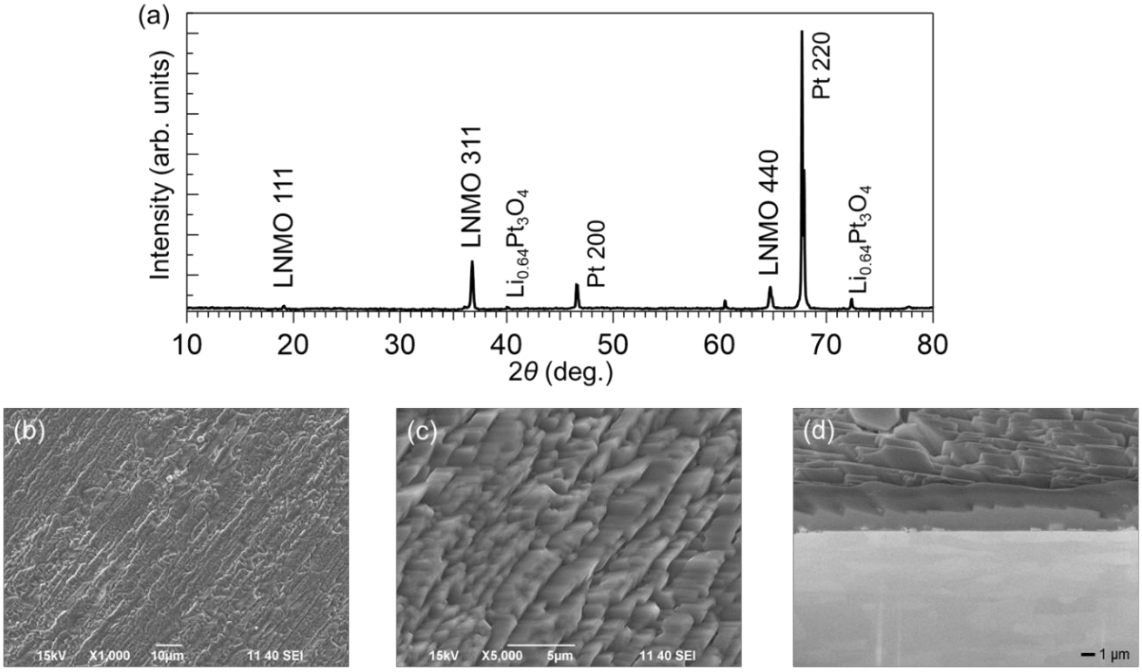

Figure S4. Structural and morphological characterizations of dense $LiNi_{0.5}Mn_{1.5}O_4$ (LNMO) layer. (a) X-ray diffraction pattern of the fabricated dense-LNMO layer. (b and c) Scanning electron microscopy (SEM) image of the layer. (d) Cross-sectional SEM image of the layer. The $Li_{0.64}Pt_3O_4$ phase is formed as a secondary phase, which is an essential finding for discussing the formation mechanism of the crystal layer. The formation of the $Li_{0.64}Pt_3O_4$ phase suggests that the Pt surface was covered with molten LiCl at an early stage of crystal growth. The reaction scheme is very similar to that of chemical vapor deposition because all the volatile raw materials are vaporized during the heating process and transported to the surface of the Pt substrate to synthesize the target compound on the surface. We note that the formation process of dense-LNMO layer surfaces with well-defined facets is controlled by liquid phase growth. Thus, it can be concluded that the raw materials were transferred into the molten LiCl–KCl covering the Pt substrate and deposited on the Pt substrate as a dense-LNMO layer.

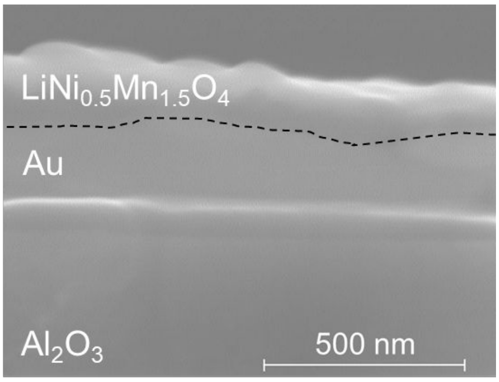

Figure S5. Cross-sectional scanning electron microscope image of thin $LiNi_{0.5}Mn_{1.5}O_4$ (LNMO) film. The LNMO thin film was deposited on the Au layer with the same deposition condition as the film on the $LaNiO_3$ current collector. Au layer was deposited on Al_2O_3(0001) substrate by DC magnetron sputtering (Au sputtering target with 1 inch diameter, output power of 20 W, Ar pressure of 0.73 Pa).

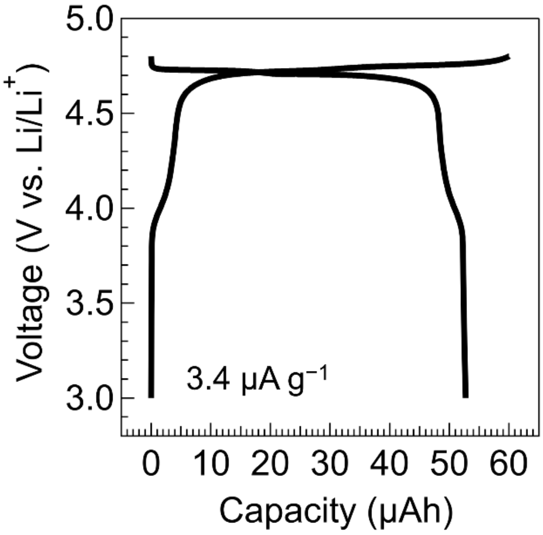

Figure S6. Galvanostatic charge–discharge curve of dense- $LiNi_{0.5}Mn_{1.5}O_4$ (LNMO) electrode-based half-cell. Cutoff voltage was in the range of 3.5-4.8 V (vs. Li/Li^+) with a 3.4 μAg^{-1} (0.2 C-rate) at 23 °C. A solution of 1 M $LiPF_6$ in a mixture of ethylene carbonate and dimethyl carbonate (1:1 vol.%) was used as the electrolyte. Li foil was used as the negative electrode.

References

1. Haruta, M.; Shiraki, S.; Ohsawa, T.; Suzuki, T.; Kumatani, A.; Takagi, Y.; Shimizu, R.; Hitosugi, T. Preparation and in-situ characterization of well-defined solid electrolyte/electrode interfaces in thin-film lithium batteries. *Solid State Ionics* 2016, 285, 118-121.

2. Kawasoko, H.; Shiraki, S.; Suzuki, T.; Shimizu, R.; Hitosugi, T. Extremely Low Resistance of Li_3PO_4 Electrolyte/$Li(Ni_{0.5}Mn_{1.5})O_4$ Electrode Interfaces. *ACS Appl. Mater. Interfaces* 2018, 10(32), 27498-27502.
